# Machine learning and engagement insights for personalized blood glucose management

**DOI:** 10.3389/fdgth.2026.1660815

**Published:** 2026-02-24

**Authors:** Inbar Breuer Asher, David L. Horwitz, Omar Manejwala, Yifat Fundoiano-Hershcovitz

**Affiliations:** 1 Dario Health, Caesarea, Israel; 2 DLH Biomedical Consulting, Las Vegas, NV, United States

**Keywords:** chronic condition, diabetes, digital health, engagement, machine learning (ML)

## Abstract

**Introduction:**

Diabetes is a chronic metabolic disorder characterized by elevated blood glucose (BG) levels, with poor control linked to serious long-term complications. Managing BG effectively requires personalized strategies, given the influence of demographic, lifestyle, and clinical factors. Machine learning (ML) offers a powerful framework for analyzing complex, real-world data to uncover individual patterns of glycemic control. Coupled with digital health platforms that enable real-time monitoring and behavioral engagement, these tools can transform diabetes care. This study leverages data from the Dario digital health platform to examine BG trends moderated by clinical and engagement variables, aiming to inform personalized digital interventions.

**Objective:**

To apply ML techniques to digital health data to identify moderating factors that influence individual BG trajectories, supporting data-driven and personalized diabetes management.

**Methods:**

A retrospective cohort study was conducted using real-world data from users with type 2 diabetes and baseline BG ≥180 mg/dL who measured BG over at least two separate months between 2020 and 2024. A piecewise linear mixed-effects model characterized BG changes over time. Generalized Linear Mixed Effects Tree models identified subgroups with distinct BG trajectories based on demographic (age, gender, BMI, ethnicity), clinical (insulin use, comorbidities, diagnosis year), and monitoring factors. An additional model tested whether lifestyle engagement (e.g., meal and activity logging) moderated BG improvement across age groups.

**Results:**

Data from 22,414 users (49.9% male; mean age 57.5; BMI 34.5) showed significant reductions in monthly average BG over 12 months (B = –6.8 in months 1–4; B = –0.3 in months 4–12; both *p* < .001). Age strongly moderated outcomes; users >60 showed the largest sustained improvements. Clinical factors such as insulin use and diagnosis duration further stratified responses, with insulin users diagnosed within five years showing the greatest reduction within the first 4 months (B = –14.3, *p* < .001). Higher frequency of BG monitoring (>12/month) was associated with greater and sustained improvements.

**Conclusion:**

Machine learning can reveal distinct glycemic trajectories moderated by demographic, clinical, and engagement factors. These findings underscore the potential of digital health platforms to personalize diabetes care and improve blood glucose management through adaptive, data-driven strategies.

## Introduction

Diabetes is a chronic disease whose complications pose a major global threat (1, 2). The rise in diabetes cases can be attributed to factors such as an aging population, poor eating habits, and a sedentary lifestyle (3, 4). Regulating blood glucose levels is essential for preventing the progression of the disease and reducing the risk of other health complications including cardiovascular disease, kidney failure, and overall decreased quality of life (5–7). Effective self-management involves controlling diet, physical activity, medication, and consistently monitoring blood glucose levels, blood pressure levels and weight (8–10). However, managing blood glucose is a complex, ongoing challenge influenced by a wide range of patient-specific factors, including demographic and medical characteristics (11–13).

The rising burden of type 2 diabetes (T2D) is a major global healthcare concern, affecting 4.4% of the population aged 15–49, 15% of those aged 50–69, and 22% of individuals aged 70 and older (14). The prevalence of T2D in the population of adults aged ≥65 is rapidly increasing worldwide due to increased life expectancy (15, 16). In addition to geriatric conditions, diabetes in older adults is associated with other age-related risk factors (17) adding to the complexity of disease management, necessitating a more personalized approach to ensure adequate glycemic control (15). Comorbidities can significantly impact a patient's ability to manage their condition (18) as they have competing demands on a patient's self-management resources (19). The presence of other medical conditions can potentially influence the type and intensity of glycemic control strategies used (20, 21), yet little is known about how they affect the patients' prioritization in diabetes management (18, 19). Additionally, factors like gender and body mass index (BMI) play a significant role in glycemic control. Research indicates that individuals with higher BMI are less likely to reach optimal glycemic targets, as obesity is linked to elevated HbA1c levels (22, 23). These findings highlight the need to account for BMI, along with other risk factors, when designing personalized management strategies.

Insulin use also impacts diabetes management. Research comparing blood glucose self-management between insulin users and non-insulin users has yielded mixed results. Studies have demonstrated that self-monitoring techniques can enhance glycemic control in insulin-treated users (24), however their impact on non-insulin-treated individuals may be less significant and often dependent on the quality of the treatment and patient education. The duration of diabetes is a key factor in how well blood glucose levels can be managed. Longer diabetes duration has been linked to poorer glycemic control and greater glycemic variability (25, 26), which is expected, considering that diabetes is a progressive condition.

Diabetes is a complex condition characterized by a wide range of clinical manifestations and varying responses to treatment, and the use of standardized treatment regimens may not fully address the unique needs and characteristics of each patient (13). Given the variability in clinical outcomes and the challenges in achieving optimal glycemic control, an individualized approach could serve as a valuable tool. A personalized approach provides an opportunity for patients to benefit from a targeted treatment (27) that takes into consideration their individual characteristics, lifestyle, and health goals, ensuring more effective management of the condition (28). Self-management is a key component of personalized care (29) as individuals are increasingly encouraged to take responsibility for their health and play an active role. However, for self-management to be truly effective, scalable and cost-effective solutions are needed to support individuals in their daily routines.

Digital health technologies have been attracting increasing attention globally in the self-management of chronic diseases (30) with mobile apps, wearable devices and digital health communities becoming more common (31, 32). Given their widespread availability, minimal barriers to access and often low cost, they have been proposed as cost-effective tools to deliver continuity of care to those who may struggle to access incumbent services (30, 33). These technologies offer great potential to provide these solutions by enabling patients to track their progress, receive feedback and encourage self-care habits on a daily basis (34).

Early efforts in digital health have focused on collecting data to track key health metrics such as blood glucose (31). Identifying meaningful patterns from the large amount of data has proven to be challenging due to the complexity and variability in individual health behaviors and conditions (35). Digital innovations offer novel opportunities to individualize a person's care to best match their lifestyle needs and circumstances and to support them as they live their daily lives with diabetes (36). The therapeutic effects are usually managed in a population-based and one-size-fits-all fashion (37) whereas the treatment efficacy generally differ between patients. However, digital data provide an excellent opportunity to use machine learning (ML) algorithms to uncover personalized treatment efficacy based on patient characteristics. Such algorithms may vary in their accuracy and clinical interpretability (38, 39).

ML has emerged as a powerful tool to address this challenge, with algorithms that are trained to detect patient-specific patterns in training data and predict future results (40). Machine learning models can assist in tailoring treatment plans to individual patients by analyzing diverse health parameters. This approach enables us to recommend personalized interventions, improving patient outcomes.

Decision trees (DTs) resemble an upside-down tree beginning with a root node out into the internal decision nodes. The process of dividing a single node into multiple nodes is known as splitting. Nodes that do not split further are called leaf or terminal nodes. Each pathway from the root to a leaf forms a branch or subtree (38, 39). DTs can model nonlinear effects and are easily interpretable if the tree depth is reasonable. DTs may help stratify patients based on their risk profiles, allowing for targeted preventive measures. By evaluating factors like age, weight, and family history, these models can predict the likelihood of developing diabetes-related complications (41, 42).

The study aims to apply ML techniques to data collected from a digital health platform to identify moderating factors that shape user-specific patterns affecting blood glucose levels. We focus on demonstrating practical implementation of analytical tools for personalized diabetes management, leveraging self-reported demographic and clinical data. By uncovering these patterns, the study seeks to generate deeper insights into the drivers of glycemic control. This personalized, data-driven approach has the potential to inform more effective management strategies and improve long-term health outcomes.

## Methods

### Platform

**Figure 1:**
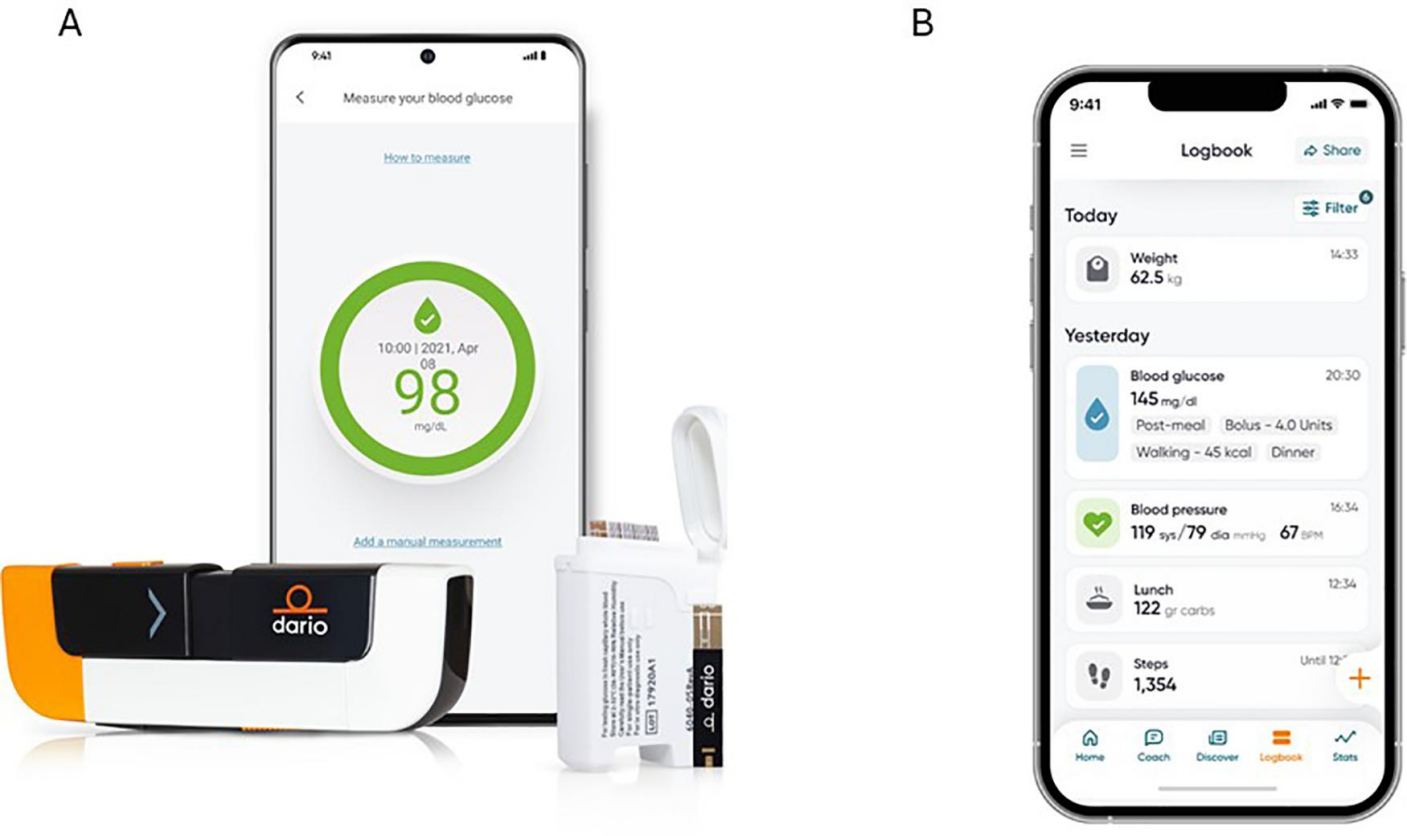
**(A)** The Dario blood glucose monitoring system, including the smartphone-connected glucose meter, test strips, and lancing device. **(B)** Example of the mobile app's logbook interface displaying daily self-monitoring data, including blood glucose readings, meals and physical activity. Images provided by DarioHealth (https://www.dariohealth.com/).

This study utilized the Dario digital health platform (Dario Health) for chronic conditions to support self-management of diabetes. The Dario platform combines a blood glucose meter with a phone app that is available for both Android and iOS devices (Figure 1). The glucose meter consists of a small, pocket-sized holder for strips, a lancet, and the meter. The blood glucose meter is removed from the holder and plugged directly into a smart mobile device, effectively converting the smart mobile device into a display screen for the meter. All data were transferred and stored in compliance with the Health Insurance Portability and Accountability Act requirements using Amazon Web Services database solutions.

### Measures

The monthly average BG (AvgBG) was defined as the means of all of a user's BG measurements taken over a 30-day interval for each user starting from the first 30 days after the initial measurement. Additionally, all collected measurements were within the valid range according to device specifications (43). It was used as the core outcome metric to reflect the monthly aggregated interval changes over 12 months. Demographic and medical information were collected for each user: Age, gender, BMI, weight, ethnicity, year of diabetes diagnosis, insulin treatment, physical activity level, stress level (0 = no stress and 10 = very stressed), alcohol consumption (number of drinks per week), smoking (0 = never and 3 = yes), and number of reported comorbidities. Digital activities were also added, such as the number of BG measurements and lifestyle tags (the sum of lifestyle activities such as: meal taggings, meal reference tagging, carbs taggings and calories burned). Those variables were analyzed vs. baseline levels in a monthly aggregation.

### Study population

The inclusion criteria were Dario platform users with reported T2D in high-risk range (44–46)

[first 30 days with AvgBG ≥180 mg/dL equivalent to A1c 8.0 (47)] who measured their BG on the Dario platform between 2020 and 2024 for a minimum of two separate months consecutive or non-consecutive (*N* = 22,414). The users purchased the device via a direct-to-consumer (D2C) channel. Exclusion criteria: registered users that did not measure BG with Dario between 2020 and 2024 for at least two separate months (insufficient data for analysis); Out of specified range measurements (43); users did not report diabetes type 2; users with AvgBG lower than 180 mg/dL in the first 30 days after the initial measurement.

### Bioethics

This study is based on an analysis of an existing database already collected in the digital platform. All data were anonymized before extraction for this study. Ethical and Independent Review Services, a U.S.-based professional review board, issued the institutional review board exemption for this study (#23517) (48).

### Study design

We applied a retrospective longitudinal cohort study design utilizing a database Dario Health D2C population. Data were obtained from the user population defined above that included their age, weight, height (BMI), duration of known diabetes and reported insulin usage.

### Analytic approach

A classical linear longitudinal model assumes a single-slope growth pattern for changes in an outcome variable across time. Sometimes, such a simple model does not fit the empirical data. In contrast, piecewise-based mixed-effects models allow exhibiting different linear trends across time. Here, based on the visualization of the distribution of the monthly average BG, which revealed a notable change in the trend around the 4-month mark a piecewise linear mixed-effects model was used to define the time-related fluctuations and allow flexibility in the modeling of variable change trajectories across time. The decision was primarily guided by the observed pattern in the visualization and supported by previous analyses conducted on the digital health platform (49, 50).

Next, a Generalized Linear Mixed Effects Tree analysis was used (51), allowing for the identification of different relationships in subgroups of data while accounting for both fixed and random effects in longitudinal data. The tree algorithm learns where each node is associated with different fixed-effects regression coefficients while adjusting for random. This allows the detection of users' subgroups with varying fixed-effects parameter estimates, keeping the random effects constant throughout the tree. The tree algorithm was incorporated with piecewise parameters to detect subgroups with different piecewise trajectories according to the potential moderating factors in 3 different models- the demographic moderating factors included age, gender, BMI and ethnicity; Medical factors included the number of comorbidities, insulin usage, BMI and year of diagnosis; and the BG monitoring moderating factor included only the monthly number of BG measurements. The grouping decision is rooted in the theoretical and clinical relevance of each factor within its respective context. The inclusion of BMI in both models is based on its distinct conceptual role: as a baseline demographic characteristic and as a current medical factor allowing us to explore its moderating effects from different perspectives. This approach may assist in identifying a specific nonlinear trajectory for the subpopulations characterized by the moderating factors.

Overfitting occurs when a machine learning model learns not only the underlying patterns in the training data but also the noise or random fluctuations, resulting in poor generalization to new, unseen data. This is a common issue, particularly with complex models relative to the amount of data available. To mitigate overfitting, the data set was randomly partitioned (by users) into 80% training set and 20% test set- which will be used to measure the predictive validity of the chosen model during the training.

All tree models are built using the lmertree function from R package “glmertree” (52, 53), under the assumption of random intercept for the users, meaning that the model considers that there is a difference in outcome between individuals. Modeling with a random intercept assumes that each individual has a unique baseline outcome, capturing inherent differences between users. This accounts for variability across individuals while focusing on the overall trajectories.

Pruning in decision tree models is a crucial technique for reducing model complexity and enhancing generalization. The pre-pruning process was deployed as all models held “minsplit” (the smallest number of observations in the parent node that could be split further) of 100, and the “maxdepth” (the maximum distance between the root and any leaf) to ensure no more than three levels of decision nodes and by that to force the model to focus on the most important splits and avoid overfitting to minor data patterns. Together, these parameters help to balance between underfitting and overfitting, ensuring that the model remains both interpretable and robust. Five-fold cross-validation for monthly average BG levels were deployed on the training data. Finally, the models were tested with an untouched (test) data set, whereas the assessment of the model's performances was evaluated with root mean square error (RMSE), comparing RMSE based on the training and test data.

## Results

In total, 22,414 users were included in this analysis. The overall cohort had a mean age of 57.5 years (SD 12.1) and was 49.9% male, with a mean body mass index (BMI) of 34.5 kg/m^2^ (SD 8.2). Participants were stratified into three age groups: ≤35 years (*n* = 856), 36–60 years (*n* = 11,550), and >60 years (*n* = 8,847).

Mean duration of diabetes at baseline was 13.5 years (SD 9.0) and increased with age, ranging from 7.8 years in users ≤35 years to 16.5 years in users >60 years. Overall, 36.4% of users were treated with insulin, with higher insulin use observed in the oldest age group. Most users had no recorded comorbidities (61.9%), although comorbidity burden increased with age.

Baseline mean blood glucose during the first month was 240.1 mg/dL (SD 59.4) and was highest among younger users. Baseline engagement during the first month was similar across age groups, with an average of approximately 20 glucose measurements per month and low levels of recorded lifestyle engagement.

Baseline demographic, clinical, and engagement characteristics for the overall cohort and by age group are presented in Table 1.

**Table 1:** Baseline demographic, clinical, and engagement characteristics of the study cohort, overall and stratified by age group.

Characteristic	Age group			
	**Overall**	**≤35**	**36–60**	**>60**
*N*	22,414	856	11,550	8,847
Age [mean (SD)]	57.53 (12.08)	30.78 (3.65)	50.83 (6.47)	68.88 (6.15)
Gender (%)				
Female	10,428 (46.5)	470 (54.9)	5,389 (46.7)	4,367 (49.4)
Male	11,183 (49.9)	386 (45.1)	6,156 (53.3)	4,465 (50.5)
Unknown	803 (3.6)	0 (0.0)	5 (0.0)	15 (0.2)
BMI [mean (SD)]	34.52 (8.20)	36.90 (9.73)	35.50 (8.51)	33.02 (7.31)
Ethnicity (%)				
Asian	183 (0.8)	12 (1.4)	115 (1.0)	55 (0.6)
Black	566 (2.5)	32 (3.7)	353 (3.1)	178 (2.0)
Latino	688 (3.1)	59 (6.9)	479 (4.1)	145 (1.6)
Other	193 (0.9)	5 (0.6)	112 (1.0)	73 (0.8)
Unknown	17,063 (76.1)	650 (75.9)	8,571 (74.2)	6,719 (75.9)
White	3,721 (16.6)	98 (11.4)	1,920 (16.6)	1,677 (19.0)
Years since diagnosis [mean (SD)]	13.48 (9.00)	7.81 (5.14)	11.55 (7.61)	16.51 (9.88)
Insulin use [Yes (%)]	8,167 (36.4)	301 (35.2)	4,157 (36.0)	3,588 (40.6)
Number of comorbidities (%)				
0	13,880 (61.9)	679 (79.3)	7,273 (63.0)	4,834 (54.6)
1	6,270 (28.0)	147 (17.2)	3,240 (28.1)	2,831 (32.0)
2	1,961 (8.7)	29 (3.4)	929 (8.0)	991 (11.2)
3+	303 (1.4)	1 (0.1)	108 (0.9)	191 (2.2)
Mean baseline month BG [mean (SD)]	240.12 (59.41)	256.41 (68.63)	242.43 (59.33)	234.54 (56.90)
Mean measurements in baseline month [mean (SD)]	19.63 (23.77)	20.07 (23.72)	19.89 (24.72)	20.02 (23.03)
Mean number of lifestyle engagements in baseline month [mean (SD)]	2.07 (12.06)	2.36 (13.49)	2.24 (11.54)	2.06 (13.26)

**Figure 2:**
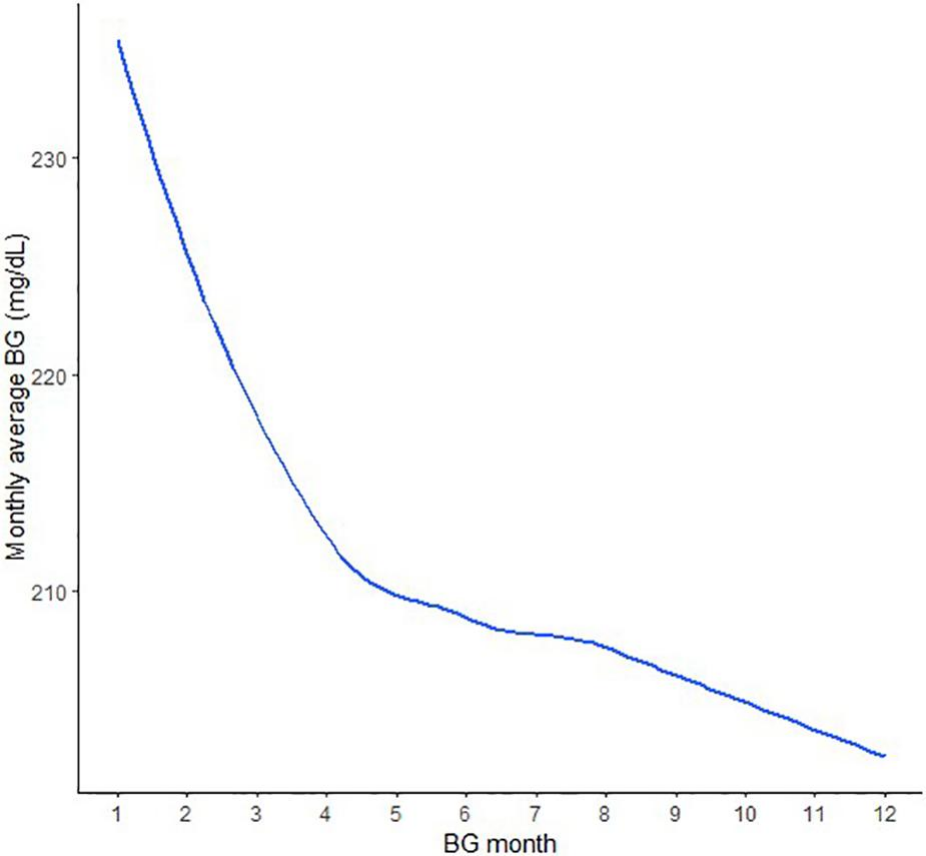
Monthly average blood glucose trajectories over a 12-month period in the overall study population.

Following a visualization of the distribution of the monthly average BG (Figure 2), a piecewise mixed model analysis was used to test the differences in the trajectories of the monthly average BG during two periods: 1–4 months and 4–12 months. A significant decrease in the monthly average BG (mg/dL) was observed in the first 4 months (B = −6.8, *p* < .001) followed by a milder decrease in the next 8 months (B = −0.3, *p* < .001); B refers to the regression coefficient, representing the average change in BG levels per month. Monthly Average BG decreased from 240.1 mg/dL in month 1 to 211.4 mg/dL in month 4, corresponding to a reduction of 28.7 mg/dL over the initial period. Between months 4 and 12, monthly average BG declined more gradually from 211.4 mg/dL to 203.0 mg/dL, representing an additional 8.4 mg/dL reduction over eight months. The confidence intervals around these estimates indicate the precision of the effect sizes, with the first 4 months showing a substantial reduction that is likely to be clinically meaningful, whereas the decline in the subsequent period is smaller but still statistically significant. This underscores the clinical importance of intervention in the first trajectory, with the smaller subsequent change indicating a potential plateau in improvement over time.

### Results of the demographic-based decision tree

**Figure 3:**
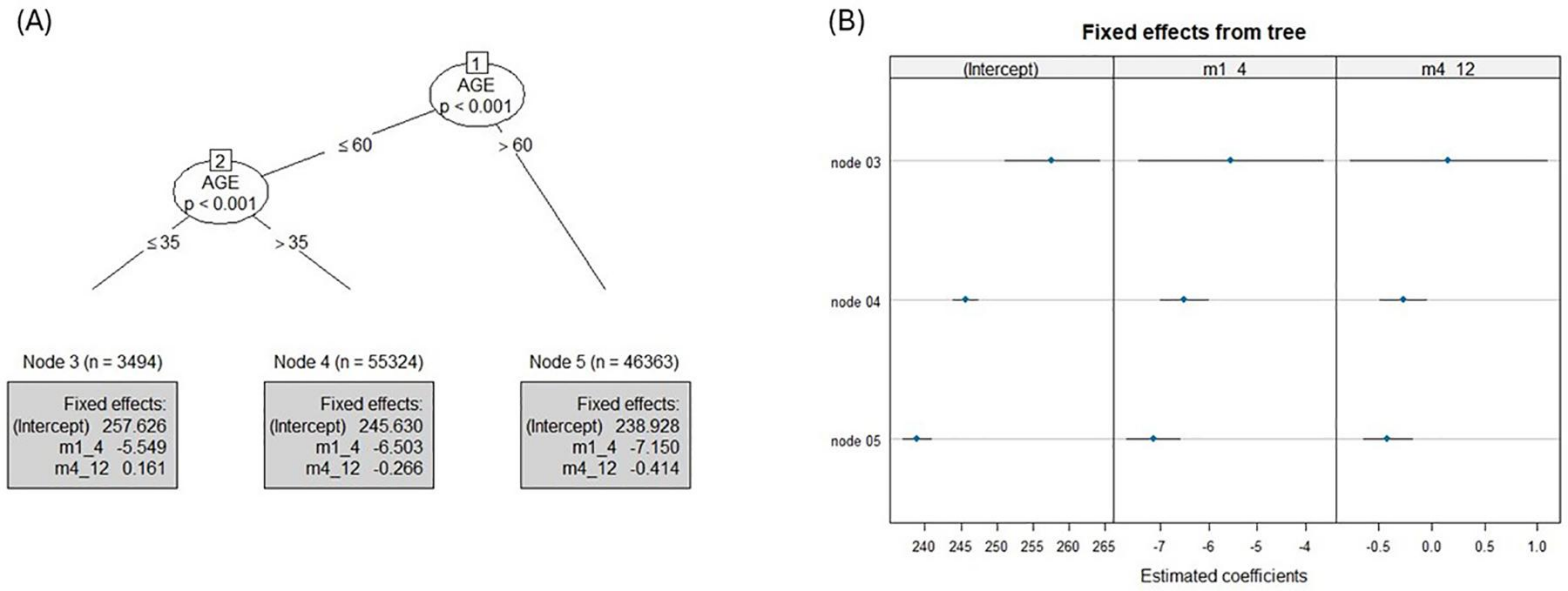
Demographic decision tree results: **(A)** piecewise mixed-effects model tree with estimated coefficients printed in the terminal nodes of the monthly average BG where the data is split into 3 age subgroups: ≤35, 36–60, >60. m1_4 represents the estimates of the first time segment (months 1–4) and m4_12 represents the estimates of the second time segment (months 4–12). **(B)** Caterpillar plot of estimated node-specific fixed effects with 95% confidence intervals for the monthly average BG, left column is the intercept and m1_4 and m4_12 represents the slopes.

Gender, BMI and ethnicity did not significantly moderate monthly average BG fluctuations. For these non-significant results, the effect sizes and confidence intervals indicate that the corresponding *P*-values are likely greater than 0.05, especially if the confidence intervals include zero. These findings highlight the broad applicability of digital health interventions across diverse demographic groups. The decision tree segmented users into three age subgroups (node 3: ≤35, node 4: 36–60, node 5: >60; Figure 3) and each exhibited a significant improvement in monthly average BG (mg/dL) in the first 4 months (node 3: B = –5.54, 95% CI −7.45 to −3.64, *p* ≤ .001; node 4: B = –6.50, 95% CI −6.99 to −6.01, *p* ≤ .001) greater in the older age group (node 5: B = –7.15, 95% CI −7.69 to −6.60, *p* ≤ .001).

Over the following 8 months, ≤35 age group showed no change in monthly average BG (node 3: B = 0.16, 95% CI −0.76 to 1.08, *p* = .74), 36–60 group had a slight reduction (node 4: B = −0.26, 95% CI −0.48 to −0.04, *p* ≤ .01) and >60 group showed the most significant improvement (node 5: B = −0.41, 95% CI −0.64 to −0.18, *p* ≤ .001).

**Figure 4:**
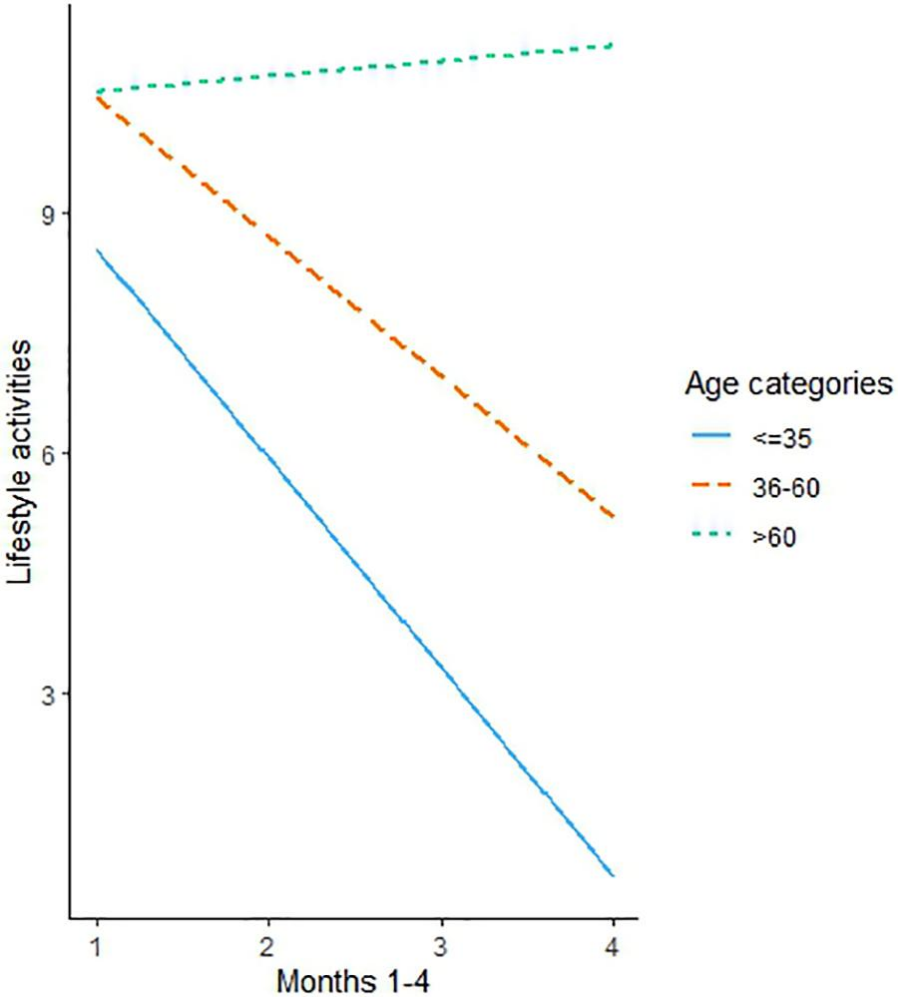
The graph presents a simple slope analysis of the lifestyle tags (activities) trajectories during months 1–4 of app use moderated by the Age segment.

Following the decision tree's results another LME model was evaluated. In this model, the interaction between the 3 age categories and lifestyle tags (the sum of meal taggings, carbs taggings and calories burned) was tested. It was found that users in the age category >60 demonstrated a greater increase in lifestyle tags during the first 4 months (B = 2.81, 95% CI 0.38 to 5.25, *p* = .02) compared to age group ≤35 (Figure 4). The interaction wasn't significant when comparing the age group of 36–60 to ≤35 (B = 0.87, 95% CI −1.54 to 3.29, *p* = .49), meaning that they were statistically equivalent.

### Results of clinical and monitoring-based decision trees

#### Clinical factors decision tree

**Figure 5:**
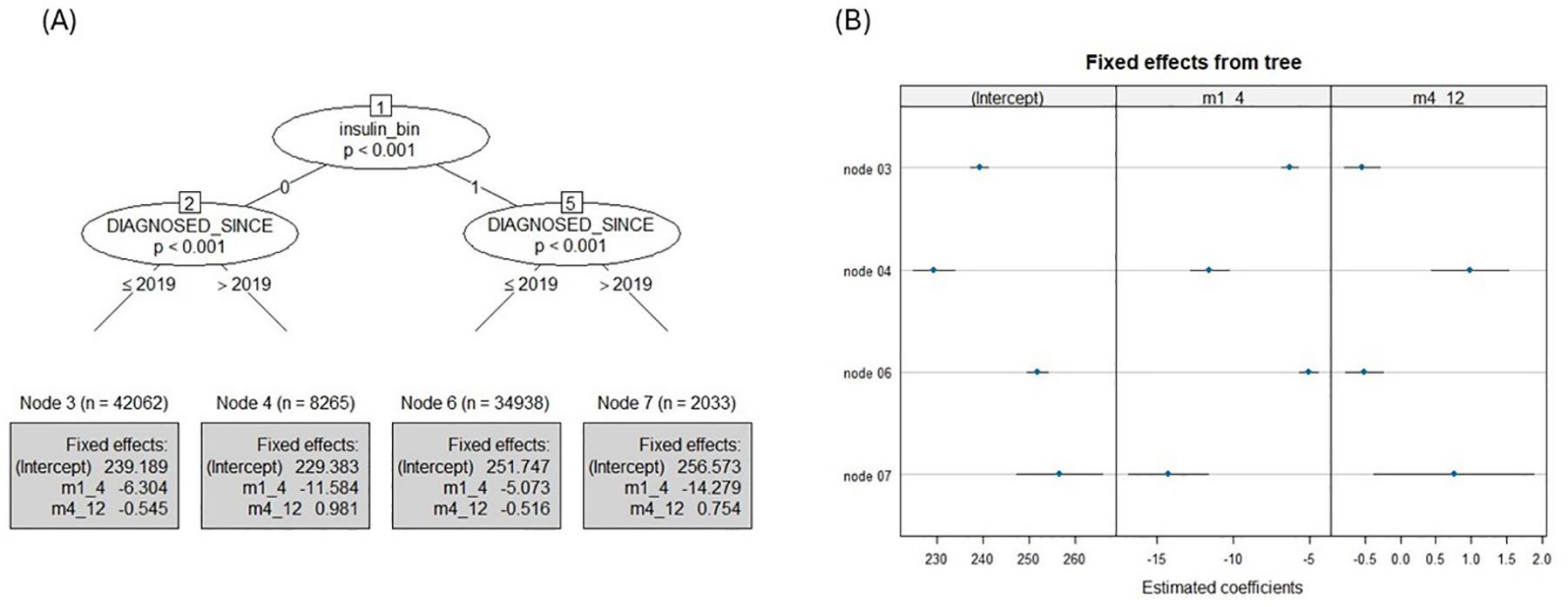
Clinical tree results: **(A)** piecewise mixed-effects model tree with estimated coefficients printed in the terminal nodes of the monthly average BG, where the data is split into 4 subgroups: the first split was for insulin users vs. non-insulin users, and the second split was for those reported diagnosis within the past 5 years (i.e., in 2019 or later) vs. those diagnosed more than 5 years ago (before 2019). m1_4 represents the estimates of the first time segment (months 1–4) and m4_12 represents the estimates of the second time segment (months 4–12). **(B)** Caterpillar plot of estimated node-specific fixed effects with 95% confidence intervals for the monthly average BG.

Figure 5 illustrates the decision tree structure and the corresponding trajectories of monthly average BG across clinical moderating factors. The first split in the tree was based on whether there is any insulin usage and then based on the year since diagnosis, categorizing users into <5 and ≥5 years (Diagnosed at 2019). In the first 4 months the monthly average BG had decreased across all groups, with the strongest effect seen in individuals using insulin and diagnosed for <5 years (node 7: B = −14.3, 95% CI −16.87 to −11.68, *p* < .001) followed by the group of users who had not been using insulin and diagnosed for <5 years (node 4: B = −11.6, 95% CI −12.86 to −10.31, *p* < .001). For individuals with a diagnosis longer than 5 years, the effect was still negative but less pronounced for both with insulin use (node 6: B = −5.1, 95% CI −5.70 to −4.45, *p* < .001) and without insulin use (node 3: B = −6.3, 95% CI −6.87 to −5.74, *p* < .001).

Regarding the second slope (months 4–12), those who were diagnosed for more than 5 years (before 2019), both with and without insulin use, had shown significant reductions in the monthly average BG (node 3: B = −0.54, 95% CI −0.79 to −0.30, *p* < .001; node 6: B = −0.51, 95% CI −0.78 to −0.25, *p* < .001). For individuals using insulin and diagnosed for less than 5 years (node 7), the second slope was not significantly associated with the monthly average BG (B = 0.75, 95% CI −0.37 to 1.88, *p* = .15), suggesting that there were no significant changes in the following months of the year. In contrast, for individuals not using insulin but diagnosed for less than 5 years (node 4), a significant increase was demonstrated in the monthly average BG during the second time segment (B = 0.98, 95% CI 0.43 to 1.53, *p* < .001).

#### Monitoring patterns decision tree

**Figure 6:**
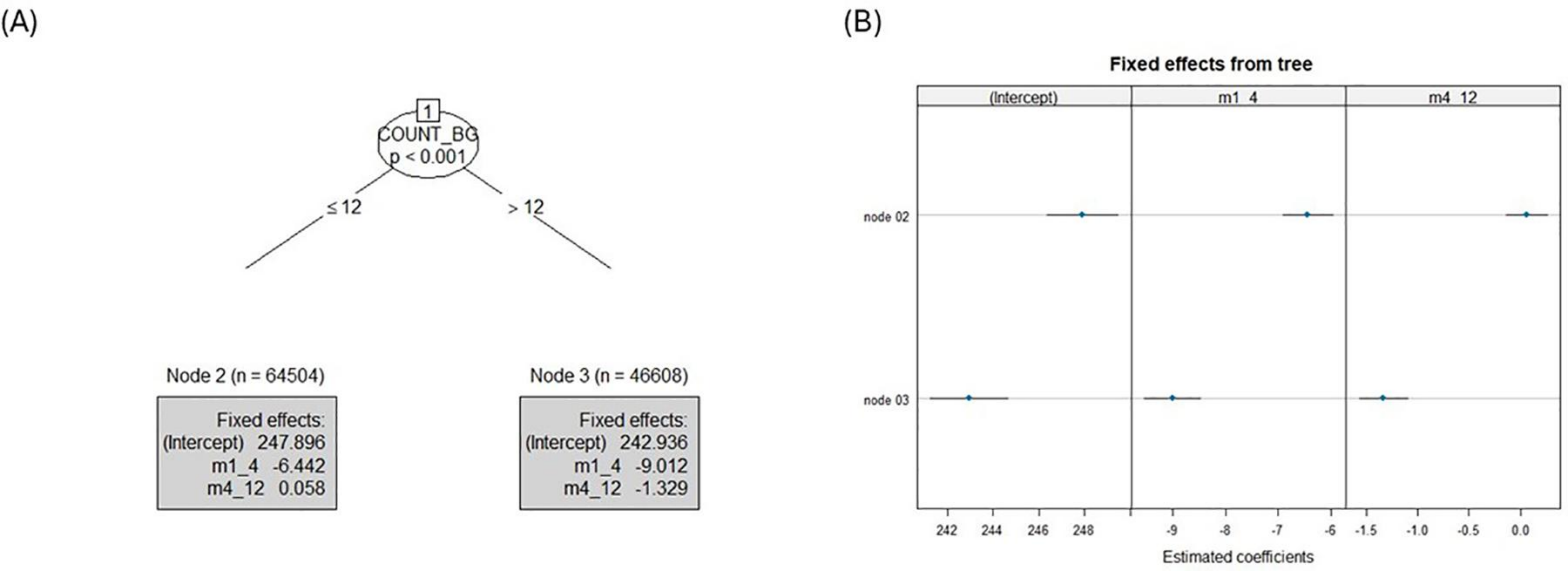
Monitoring tree results: **(A)** Piecewise mixed-effects model tree with estimated coefficients printed in the terminal nodes of the monthly average BG, where the data is split into two COUNT_BG groups: individuals with ≤12 measurements and those with >12 measurements. m1_4 represents the estimates of the first time segment (months 1–4) and m4_12 represents the estimates of the second time segment (months 4–12). **(B)** Caterpillar plot of estimated node-specific fixed effects with 95% confidence intervals for the monthly average BG.

Figure 6 presents the decision tree and the corresponding changes in monthly average BG stratified by the number of monthly BG measurements. The following model divided the number of monthly BG measurements into 2 segments, based on a cutoff of 12 measurements, one group with number of monthly BG measurements ≤12 (node 2) and another group with >12 (node 3). The results show that in the first 4 months, both groups had significant reductions in the monthly average BG, yet for individuals with monthly number of BG measurements >12, a stronger reduction was demonstrated (node 3: B = −9.01, 95% CI −9.54 to −8.48, *p* < .001; node 2: B = −6.44, 95% CI −6.92 to −5.97, *p* < .001).

In the second segment, individuals with monthly number of BG measurements >12 had shown further significant reductions in the monthly average BG, yet weaker than the first period (node 3: B = −1.33, 95% CI −1.57 to −1.09, *p* < .001). In contrast, for individuals with ≤12 monthly BG measurements, the monthly average BG did not change significantly (node 2: B = 0.06, 95% CI −0.14 to 0.26, *p* = .57).

To assess whether the likelihood of having a blood glucose measurement at month 12 differed across key demographic and clinical subgroups, we examined measurement availability stratified by age, insulin use, and diabetes duration. Among users younger than 60 years, 31.8% had a measurement at month 12, compared with 38.5% of users aged 60 years or older. Measurement rates were comparable across diabetes duration groups, with 34.9% of users diagnosed for <5 years and 34.6% of those diagnosed for ≥5 years contributing 12-month data. Insulin users were slightly more likely to have a month-12 measurement than non-insulin users (37.0% vs. 32.8%). Overall, these subgroup differences were modest and did not suggest systematic exclusion of any demographic or clinical group from later follow-up assessments.

### Sensitivity analysis

To assess the robustness of our findings to key modeling assumptions, we conducted comprehensive sensitivity analyses testing alternative model specifications, data inclusion criteria, and analytical approaches. We tested alternative piecewise breakpoints (months 3, 4, 5, 6), different age and BMI modeling approaches (continuous, quartiles, categories), outlier removal (excluding BG >500 or <50 mg/dL), consecutive vs. non-consecutive months, different baseline BG levels, and different training/test splits (70/30 to 85/15). The 4-month breakpoint (months 1–4) provided the best model fit among the tested alternatives (lowest AIC score), confirming this as the best-fitting specification (slope months 1–4: −6.80 mg/dL/month, *p* < .001; slope months 4–12: −0.33 mg/dL/month, *p* < .001). Alternative breakpoints (3, 5, 6 months) also showed statistically significant results for months 1–4, but with inferior model fit. Age was remodeled into categories (≤35, 36–60, ≥60) and quartiles. All age groups showed similar improvements during months 1–4 across different age modeling approaches, indicating that initial response was consistent regardless of age. Age significantly moderated sustained improvements during months 4–12 (quartiles: B = −0.423, *p* = 0.041; categories: >60 years, B = −0.41, *p* < .001), with older users showing greater sustained improvements, validating the decision tree findings. BMI was remodeled into categories (Normal<25, Overweight 25–30, Obese I 30–35, Obese II 35–40, Obese III >40). Results showed similar improvements in all categories for both time periods (all *p* > 0.05).

Results were highly consistent across alternative data inclusion criteria and model specifications. Outlier removal (excluding 0.9% of data) produced nearly identical estimates (both segments *p* < .001). Analyses restricted to consecutive months only (*n* = 9,887 users) showed stronger but consistent effects (months 1–4: −8.81 mg/dL/month; months 4–12: −0.80 mg/dL/month, both *p* < .001), suggesting that sustained engagement is associated with better outcomes. When testing different training/test splits (70/30 to 85/15), slope estimates for months 1–4 ranged from −6.64 to −6.82 mg/dL/month (all *p* < .001), and test RMSE remained stable (73.1–73.5 mg/dL).

Results remained statistically significant and directionally consistent across all alternative specifications, demonstrating that conclusions are not dependent on specific modeling choices or data inclusion criteria. The pattern of stronger effects in more engaged users (those with consecutive data) is expected and clinically meaningful, as it suggests that sustained engagement is associated with better outcomes. This pattern aligns with our primary finding that monitoring frequency moderates outcomes.

## Discussion

### Principal results

The study leveraged ML methods to identify user-level factors influencing blood glucose levels among people managing diabetes using digital health platforms. A significant reduction in monthly average BG was observed during the first four months of engagement, with a milder decrease in subsequent months.

The magnitude of blood glucose reductions observed in this study is clinically meaningful and comparable to established benchmarks for behavioral and pharmacologic interventions. During months 1–4, mean blood glucose declined by 28.7 mg/dL, which corresponds to an estimated reduction of approximately 1.0% in HbA1c, based on validated conversion formulas (47). Reductions of this size are similar to those achieved with first-line glucose-lowering therapies in early treatment escalation (54, 55–57). Even the smaller additional decline of 8.4 mg/dL observed during months 4–12 corresponds to an estimated 0.3% reduction in HbA1c, which remains clinically significant. Evidence from large cohort studies and meta-analyses demonstrates that HbA1c improvements as modest as 0.3%–0.5% are associated with meaningful reductions in microvascular complications, improved treatment stability, and lower healthcare utilization (58, 59). Importantly, the sustained improvement during months 4–12 reflects maintenance rather than regression an imperative outcome in diabetes management, as behavioral benefits often diminish over time in real-world settings. These outcomes reinforce the potential of digital behavioral interventions to deliver health benefits comparable to established therapeutic approaches and highlight their value as an adjunct to routine diabetes care (60, 61).

The findings highlight how user characteristics such as age, insulin use, disease duration, and frequency of blood glucose self-monitoring can moderate glycemic changes over time. These factors, in combination with lifestyle tags that reflect digital engagement, interact to influence overall outcomes. Particularly, older adults (>60 years), users with frequent BG monitoring (>12 times/month), and those using insulin with a shorter disease duration (<5 years) showed the most substantial improvements in BG control.

Our results highlight two critical insights. First, the most substantial improvement in average BG occurred during the first four months of platform use across all user subgroups, consistent with earlier findings showing an initial engagement-driven benefit from digital self-monitoring tools (8, 10). Second, older adults (≥60 years) and users who engaged more consistently in lifestyle tags within the app such as meal tagging, meal reference tagging, carbohydrate tagging, and tracking calories burned, showed more sustained improvements in blood glucose throughout the year. This suggests that consistent self-monitoring is a key factor in long-term glycemic management.

#### Comparison to prior work

One of the results highlights is that the most substantial improvement in average BG occurred during the first four months of platform use across all user subgroups, consistent with earlier findings showing an initial engagement-driven benefit from digital self-monitoring tools (8, 10, 50). This work reinforces the importance of sustained engagement to prolong clinical benefits. The piecewise-based model indicated users observed a significant decrease in BG levels during the initial 4 months of platform usage, followed by a stable or mild-improvement period during months 4–12 of the monitoring. Consistent with previous studies, we found that users of a connected glucose monitor experienced the most change in their first few months of use (49, 62, 63). Of note, change patterns with an early rapid change period followed by a long-tailed period where change is retained appeared in many real-world digital interventions for behavior change (64).

Contrary to prior studies suggesting that BMI and ethnicity may influence glycemic control (23, 65), our findings did not identify a significant moderating effect of these variables on monthly average blood glucose fluctuations. The absence of significant moderation by BMI suggests that digital intervention may be equally effective across a wide range of BMI and body compositions. The finding that ethnicity did not moderate BG outcomes may point to the broad applicability and generalizability of the digital health approach. Ethnic disparities in diabetes outcomes are well documented (66), often driven by differences in access, education, cultural norms, and systemic factors. However, digital interventions, may help bridge these gaps, offering consistent and personalized support regardless of background (67). This supports the value of digital tools as counterbalances, delivering effective support across users with varying physical profiles (67, 68).

The decision tree analyses provided key insights into the heterogeneous response to digital diabetes self-management. Older adults (≥60 years) and users who engaged in more frequent monitoring or lifestyle tracking activities exhibited more sustained BG improvements throughout the year, suggesting that consistent self-monitoring plays a crucial role in long-term glycemic management. Age-related differences echo findings from previous studies, indicating that older adults may be more consistent in self-care behaviors, adherence to chronic illness self-care and more responsive to structured interventions (69, 70). Increases in lifestyle tags were also most pronounced in this group, supporting the idea that older adults may be particularly responsive to behaviorally driven interventions when appropriately designed (71). Lifestyle outcomes, including weight loss and increased physical activity were observed primarily in middle to older aged adults with intervention durations of longer than 12 weeks proving most effective (71, 72). In contrast, younger users (≤35 years) had limited sustained improvement, underscoring the need for targeted re-engagement strategies in this subgroup.

The clinical based decision tree, stratified by insulin use and diabetes duration, emphasized the importance of disease progression and treatment intensity in diabetes management. Particularly, users with insulin therapy and recent diagnoses (<5 years) experienced the greatest reductions in average blood glucose during the first four months. This may reflect clinical responsiveness and motivation among individuals newly initiated on insulin, who are likely more engaged with their self-care routine and responsive to digital support (73, 74). Conversely, among users with longer disease duration (≥5 years), glycemic improvements were more modest, suggesting that long-standing diabetes may be associated with physiological resistance to change or diminished beta-cell function, limiting further glycemic improvement despite digital engagement (75, 76).

Regarding the second slope (months 4–12), those who were diagnosed for more than 5 years, both with and without insulin use, had shown significant reductions in the monthly average BG. Interestingly, among non-insulin users diagnosed within the past 5 years, an increase in average BG was observed during this second period. This deterioration may indicate an unmet need for treatment intensification in this subgroup, as the initial benefits of engagement could diminish without ongoing pharmacological support.

Several subgroup patterns in this analysis warrant additional consideration. Although the months 4–12 slopes for users aged ≤35 years and for non-insulin users diagnosed within the past five years were not statistically significant, this likely reflects limited precision due to smaller subgroup sizes and substantial month-to-month variability rather than a true absence of effect. Younger adults with type 2 diabetes often demonstrate lower adherence to self-management behaviors, reduced monitoring frequency, and less consistent engagement with digital tools, which may attenuate long-term intervention effects. Prior research suggests that this population may benefit from more tailored engagement strategies, such as enhanced personalization or motivational supports (77–79).

The increase in blood glucose observed among recently diagnosed non-insulin users from months 4–12 is clinically important and consistent with the early progression of type 2 diabetes, where declining β-cell function and delayed treatment intensification can lead to worsening glycemic control (80). Current clinical guidelines emphasize the need for timely reassessment when rising glucose trends are detected, highlighting the importance of integrating triage pathways within digital programs to prompt users to seek provider evaluation (81).

These findings underscore the need for adaptive intervention approaches that account for demographic and clinical differences, including dynamic personalization, alerts for concerning glycemic trajectories, and alignment with guideline-based treatment escalation. Future work should evaluate whether tailored or hybrid digital-clinical strategies can better support these higher-risk subgroups.

The monitoring-based decision tree demonstrated that blood glucose measurement frequency is a strong moderator of blood glucose levels. Users who measured their BG more than 12 times per month showed significantly greater and sustained reductions in average BG during both the initial 4-month and subsequent 8-month periods. In contrast, users with lower measurement frequency (≤12 times/month) experienced initial improvements that were not sustained beyond the early phase. This pattern reinforces existing evidence that frequent self-monitoring of blood glucose (SMBG) is not only a marker of engagement but also a driver of glycemic control, possibly due to increased awareness, better adherence, and timely behavioral adjustments (82–84). These results suggest that encouraging more frequent SMBG, particularly in the first months of platform use, may optimize the clinical benefit of digital interventions. It is particularly interesting to observe that the machine learning analysis of real-world data identified 12 blood glucose measurements per month as a meaningful cut-off point distinguishing different glycemic trajectories. While no strict monthly minimum is defined for all patients, limited but regular monitoring (e.g., few times per week) is commonly advised for people with type 2 not on insulin. This results in approximately 8–16 checks per month, closely matching the >12/month threshold in our analysis (85, 86).

This study found that the digital health platform was more effective for certain groups during diabetes management, raising the question of whether programs should be adapted to better support younger adults. The insights point to the need for personalized feedback loops, users demonstrating high measurement frequency may benefit from positive reinforcement and long-term goal setting, while those with lower frequency may require nudges, behavioral activation, or structured reminders to sustain their engagement. Moreover, this approach can be used to assess whether other developed features or programs demonstrate efficacy during product usage.

In real-world digital health research, engagement patterns and self-monitoring behaviors naturally fluctuate over time. The level of data availability in our study is relatively high compared with typical patterns reported in similar real-world digital health studies, which often show 12-month measurement rates in the range of 20%–40% (87, 88).

These findings highlight the importance of personalized follow-up strategies. Digital platforms should dynamically adjust content and support frequency based on a user's treatment stage and disease progression, particularly for those at risk of glycemic rebound. This approach aligns with the principles of personalized medicine, which emphasize that individual users differ across sociodemographic, physiological, and behavioral dimensions, and therefore may benefit from tailored interventions. The goal is to identify data patterns that align with known subgroups, such as the presence of moderating factors influencing treatment response and outcomes.

### Limitations

This study is subject to several limitations. The observational design limits causal inference: associations between engagement patterns and BG improvements may be influenced by unmeasured confounders such as user motivation or socioeconomic status. Although the models included key demographic and behavioral variables, psychosocial factors such as mental health status, digital literacy, or readiness for change were not available and could also impact the results. Selection bias may also be present, as the sample consists of direct-to-consumer (D2C) users who voluntarily opted into the program, potentially limiting to broader patient populations. Additionally, the absence of clinical gold-standard measures such as HbA1c limits our ability to assess glycemic control based on laboratory tests. Attrition bias is another concern, as participants with incomplete data may differ systematically from those who remained engaged, influencing the observed outcomes. In the present study, more than one-third of users had available measurements at month 12, a level of data availability comparable to that reported in prior long-term digital and mobile health studies. In addition, sensitivity analyses demonstrated that the primary findings were robust to alternative data inclusion criteria. Observational nature introduces potential temporal confounding, where changes over time may be influenced by external factors unrelated to the intervention.

The absence of a control group limits comparisons, making it difficult to attribute outcomes solely to the intervention rather than natural disease progression or external influences. Lastly, potential multicollinearity among several predictors in the machine learning models should be acknowledged. Factors such as age, insulin use, diabetes duration, BMI, and engagement frequency are known to be interrelated in the context of diabetes management. For example, longer disease duration is often associated with a higher likelihood of insulin therapy, and younger age groups may demonstrate different engagement patterns. ML approaches such as tree-based models used in this analysis are generally robust to correlated predictors, as they split the data using the most informative variables and assess each feature's contribution through hierarchical partitioning rather than assuming linear independence. Future studies with larger samples and explicit modeling of interaction terms, or dimensionality-reduction techniques, may help clarify the independent vs. synergistic contributions of these variables.

## Data Availability

The datasets presented in this article are not readily available due to company privacy policy but are available from the corresponding author on reasonable request according to the subject to company policies. Requests to access the datasets should be directed to yifat@dariohealth.com.
